# A Portable Electrical Response Verification System for Blood Glucose Meters Based on Dynamic Microcurrent Reproduction

**DOI:** 10.3390/s26185895

**Published:** 2026-09-17

**Authors:** Wenshu Yue, Yifan Li, Jinhui Cai

**Affiliations:** College of Metrology Measurement and Instrument, China Jiliang University, Hangzhou 310018, China; p24020854146@cjlu.edu.cn (W.Y.);

**Keywords:** blood glucose meter, programmable current source, electrical response verification, dynamic microcurrent reproduction, DAC code table

## Abstract

**Highlights:**

**What are the main findings?**

- A portable electrical response verification system based on dynamic microcurrent reproduction was developed using an experimentally calibrated programmable microampere-level current source.
- The reproduced waveforms showed mean Pearson correlation coefficients of 0.9993–0.9995 and peak current CV values of 1.59–2.52%. The corresponding meter responses showed relative differences of 1.18–1.70% and CV values of 1.29–1.81%.

**What are the implications of the main findings?**

- Once the level-specific reference waveforms and corresponding DAC code tables have been established, the same characterized electrical stimulus can be repeatedly applied for controlled bench top verification.
- The proposed method provides a supplementary approach for controlled electrical response verification and short-term repeatability assessment of strip-based blood glucose meters.

**Abstract:**

Portable blood glucose meters are commonly evaluated using test strips and control solutions, which assess the combined meter–strip–reagent response but are not designed to provide a directly controlled, pre-characterized electrical stimulus for repeated meter-side verification. This study developed a portable electrical response verification system based on dynamic microcurrent reproduction using a calibrated programmable current source. At each of two control solution levels, five 1000-point test strip current responses were acquired. Each response was reduced to 100 points by averaging every 10 consecutive samples, and the five reduced responses were then averaged point by point to obtain one level-specific mean reference waveform. Each mean waveform was converted into a 100-point DAC code table for STM32-controlled reproduction. The current source provided a measured output range of 0.016–9.829 μA. Five repeated reproductions per level yielded mean Pearson correlation coefficients of 0.9993 and 0.9995 and peak current CV values of 2.52% and 1.59%, respectively. The corresponding GA-3 meter (Sinocare Inc., Changsha, Hunan, China) readings differed from the mean original readings by 1.70% and 1.18%, with CV values of 1.81% and 1.29%. These results support the feasibility of controlled electrical response verification and short-term repeatability assessment of strip-based blood glucose meters.

## 1. Introduction

Diabetes represents a major and growing global health burden [1], and regular glucose assessment plays an important role in diagnosis and daily disease management. Strip-based portable blood glucose meters remain widely used for self-monitoring of blood glucose (SMBG) and point-of-care testing because of their simplicity, rapid response, and low instrumentation requirements [2]. Meanwhile, continuous glucose monitoring (CGM) systems have become increasingly important because they provide continuous or near-continuous information on glucose trends, whereas conventional finger prick measurements provide intermittent glucose readings [3]. Thus, CGM and strip-based blood glucose meters address different monitoring needs. The present study does not aim to replace CGM or routine patient glucose monitoring but instead focuses on controlled electrical response verification of strip-based blood glucose meters.

In routine SMBG, measurement reliability can be affected by user- and sample-related factors, including insufficient blood volume, fingertip contamination, inappropriate hand washing, and improper handling or storage of test strips [4]. These practical sources of error are important in daily glucose testing but are outside of the scope of the present study. Accordingly, this work does not regard fluctuations in test strip current as a dominant cause of inaccurate glucose measurements. Instead, the time-varying current generated by the test strip is treated as an electrical input to be reproduced under controlled conditions for evaluating the meter’s electrical input–response path.

Portable blood glucose meters are commonly checked using matched test strips and manufacturer-provided control solutions [5]. Control solution testing evaluates the combined response of the meter, test strip, reagent system, and operating procedure and remains necessary when overall system performance is to be assessed. However, each control solution test involves a new strip/reagent reaction and is intended to evaluate the combined meter–strip–reagent response rather than providing a directly controlled, pre-characterized electrical stimulus for repeated meter-side bench testing. A programmable method capable of reproducing a previously characterized test strip current–time response may therefore provide a complementary tool when the specific objective is controlled evaluation of the meter’s electrical input–response path.

Electrochemical blood glucose meters commonly employ amperometric detection in which glucose-related reactions on the test strip generate a weak electrical current that is detected by the meter and converted into a displayed glucose result [6]. The resulting test strip current response is time-dependent rather than constant and exhibits characteristic transient behavior during the measurement process. Consequently, a single fixed-current stimulus cannot fully represent the temporal profile of the electrical input presented to the meter. Dynamic reproduction of the measured current–time response may therefore provide a more representative electrical stimulus for controlled verification of meter response consistency. Programmable voltage-to-current conversion based on operational amplifier circuits provides a practical means of generating such controlled microampere-level currents [7].

Based on this principle, the present study developed a portable electrical response verification system consisting of an STM32F103RCT6 microcontroller (STMicroelectronics, Geneva, Switzerland), a digital-to-analog converter (DAC), and a programmable microampere-level constant current source. Experimentally acquired test strip responses were converted into level-specific DAC code tables for controlled dynamic microcurrent reproduction.

The research question addressed in this study is whether an experimentally characterized, time-varying test strip current response can be converted into a controlled and repeatable electrical stimulus for evaluating the meter-side electrical input–response path. The contribution of the proposed system is therefore not a new glucose measurement principle but a bench top verification capability that enables a characterized dynamic electrical stimulus to be repeatedly applied to the meter input under controlled conditions. Accordingly, the intended application of the proposed system is bench top engineering and laboratory verification rather than routine patient glucose monitoring. Potential users include blood glucose meter developers, engineering laboratories, and quality control personnel who need to repeatedly evaluate meter-side electrical responses under controlled conditions. Once a level-specific mean reference waveform and its corresponding DAC code table have been established, the same characterized electrical stimulus can be repeatedly applied without requiring a new test strip/control solution reaction for every subsequent bench top electrical test. The practical benefit therefore lies in the controllability and repeatability of the applied electrical input and enabling meter-side response to be examined separately from a new strip/reagent reaction, rather than reducing test strip use during routine patient monitoring. The proposed method is intended to complement, rather than replace, conventional control solution testing or reference-based analytical evaluation, and it does not establish metrological traceability to an independent glucose reference.

The main contributions of this study are as follows:

(1) A portable electrical response verification method was developed to reproduce the measured time-varying current responses of glucose test strips using an experimentally calibrated programmable microampere-level current source.
(2) A level-specific waveform-to-DAC-code conversion procedure was established. For each control solution level, five 1000-point current responses were reduced individually to 100 points by 10-point averaging and then averaged point by point to obtain one level-specific mean reference waveform. The resulting waveform was converted into an executable DAC code table based on the experimentally measured DAC code–current relationship.
(3) Each level-specific mean reference waveform was reproduced in five repeated runs using the same DAC code table. The resulting output waveforms were used to evaluate reproduction agreement and repeatability, while the corresponding GA-3 meter readings were used to evaluate short-term meter response repeatability.

## 2. Materials and Methods

### 2.1. Principle of Electrical Response Verification of Blood Glucose Meters

Portable electrochemical blood glucose meters determine glucose concentration from the current response of disposable blood glucose test strips [8]. During measurement, glucose reacts with the enzyme reagent immobilized on the test strip, and the resulting electron transfer process produces a weak electrochemical current at the electrode interface [9]. From the perspective of the current measurement circuit, the glucose-related reaction current considered in this study flows through the principal path formed by the working electrode and the counter electrode [10]. Experimental studies of amperometric glucose sensors have shown that the generated current varies systematically with glucose concentration [11]. The blood glucose meter detects the electrical signal generated by the test strip and converts it into a displayed glucose result [12,13].

**Figure 1:**
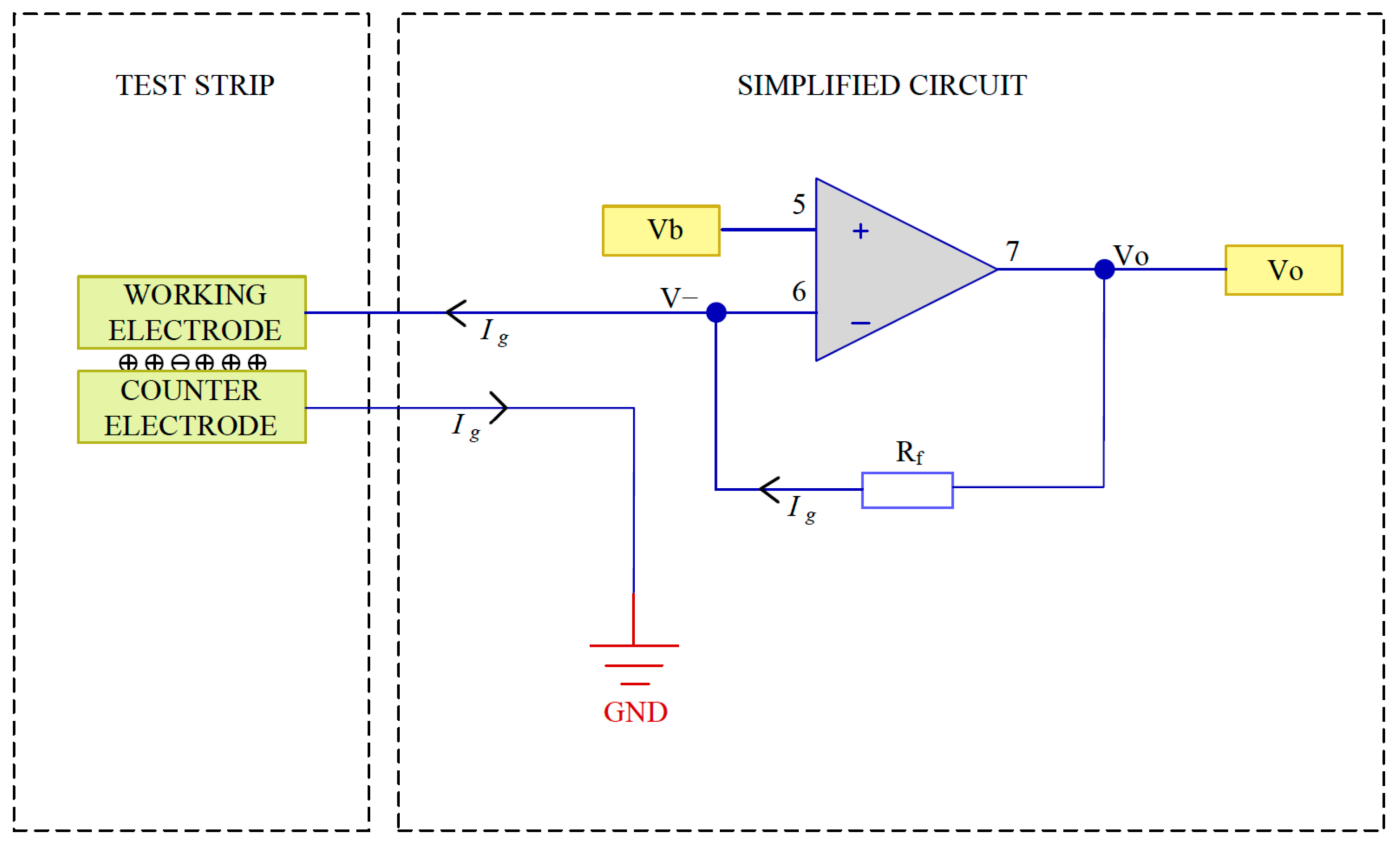
Simplified signal detection principle of a portable blood glucose meter showing the principal glucose-related current measurement path through the working and counter electrodes.

The GA-3 test strip used in this study contains three electrodes. In the present circuit analysis, only the working and counter electrodes involved in the measured glucose-related current path are considered. Therefore, for clarity, only these two electrodes are shown in Figure 1. The current signal at the test strip interface serves as the primary electrical input considered for blood glucose measurement in this work.

The manufacturer specifies a blood glucose measurement time of approximately 10 ± 1 s for the GA-3 meter [14]. However, the manufacturer’s instructions for use do not disclose which current-derived features are used or how the internal signal processing algorithm calculates the displayed glucose value. Therefore, the present study reproduced the principal temporal characteristics of the measured current response rather than assuming that a particular single current value is used by the meter.

As shown in Figure 1, the blood glucose meter applies a bias voltage $V_{b}$ and measures the glucose-related electrochemical current $I_{g}$ generated by the test strip. Because the input current of the operational amplifier is negligible, the same current $I_{g}$ flows through the feedback resistor $R_{f}$, producing the corresponding output voltage $V_{o}$. Based on this principle, a DAC-controlled microampere-level constant current source was used to reproduce the amplitude and temporal profile of the measured test strip current response.

### 2.2. Reference Waveform Acquisition and DAC Code Table Generation

To obtain reference waveforms for dynamic current reproduction and evaluate the repeatability of the test strip current responses, measurements were performed using a Sinocare GA-3 portable blood glucose meter (Sinocare Inc., Changsha, Hunan, China), by matching three-electrode test strips (Sinocare Inc., Changsha, Hunan, China; lot No. 631221), and manufacturer-provided blood glucose control solutions (Sinocare Inc., Changsha, Hunan, China; lot No. 61A4091). According to the manufacturer’s instructions, the control solution contained 5% viscosity modifier, 0.2% preservative, 0.1% colorant, 0.01 mol/L phosphate buffer, and glucose at two levels [15]. The manufacturer-specified control ranges were 3.5–5.3 mmol/L (63.0–95.4 mg/dL) for level I and 8.2–12.3 mmol/L (147.6–221.4 mg/dL) for level II. The control solutions were formulated glucose reagents rather than blood samples and used without additional dilution or preparation.

According to the manufacturer’s instructions for the GA-3 meter, the specified accuracy requirement is within ± 0.83 mmol/L (±15 mg/dL) at glucose concentrations ≤ 4.2 mmol/L (≤75 mg/dL) and within ±20% at concentrations > 4.2 mmol/L (>75 mg/dL) [14]. Because all meter readings in this study were above 4.2 mmol/L, the applicable requirement was ±20%.

For each control solution level, five independent measurements were performed under the same experimental conditions. For each measurement, both the 1000-point current–time response and the corresponding GA-3 meter reading were recorded. Measurements obtained within the same control solution level were treated as replicates rather than measurements representing distinct glucose concentrations. For each measurement, a new test strip was inserted into the meter, and approximately 0.6 μL of the corresponding control solution was applied to the sample inlet in accordance with the manufacturer’s instructions [16]. A bias voltage of 0.3 V was applied between the working electrode and the counter electrode. The current was measured using a Keysight 34470A seven-and-a-half-digit digital multimeter (Keysight Technologies, Santa Rosa, CA, USA) connected in series in the electrode–current path, with its positive input connected to the working electrode side and its negative input connected to the counter electrode side. The sampling rate was set to 100 Hz, corresponding to a sampling interval of 10 ms. Each measurement lasted 10 s and therefore produced 1000 current samples. All measurements were performed under ambient laboratory conditions.

The measured current–time responses were used to evaluate the repeatability of the test strip electrical response.

**Figure 2:**
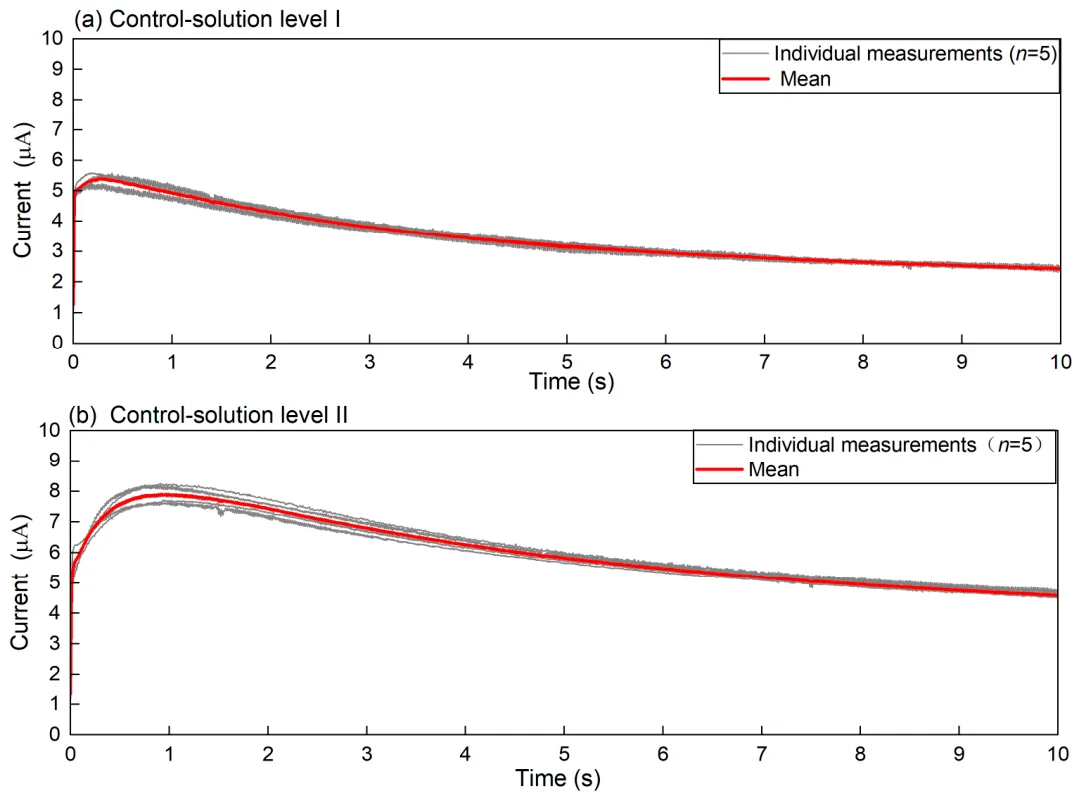
Repeatability of the measured current–time responses obtained from five independent measurements at two control solution levels: (**a**) control solution level I and (**b**) control solution level II. Gray curves represent the individual measurements (n = 5), and the red curve represents the mean response.

The measured current responses are shown in Figure 2. For both control solution levels, the current increased rapidly after sample application, reached a peak, and subsequently decreased toward a relatively stable value. The five repeated measurements within each level exhibited similar temporal characteristics, while level II generally showed higher current amplitudes than level I. The mean peak currents were 5.475 ± 0.139 μA for level I and 7.936 ± 0.277 μA for level II, corresponding to CV values of 2.55% and 3.50%, respectively, indicating good repeatability of the measured test strip current responses. The five corresponding GA-3 meter readings obtained during the original control solution measurements were averaged separately to obtain the mean original meter reading for each control solution level, which was later used for comparison in the meter response evaluation.

To reduce the number of data points while retaining the main dynamic characteristics, each of the five 1000-point current–time responses obtained at a given control solution level was divided into 100 non-overlapping groups, with 10 consecutive samples in each group. The arithmetic mean of each group was first calculated for each individual response. The five reduced values corresponding to the same group were then averaged across the five replicate measurements to obtain one target current point:

(1) $$I_{t\arg et}(k)=\frac{1}{5}\sum_{r=1}^{5}\left\{\frac{1}{10}\sum_{j=1}^{10}I_{r}[10(k-1)+j]\right\},\;\;k=1,2,\ldots ,100$$

where $I_{r}\left[n\right]$ denotes the n-th current sample of the r-th original current–time response, r=1,2,…,5 indexes the five replicate measurements, j=1,2,…10 indexes the samples within each group, and $I_{t\arg et}(k)$ denotes the k-th point of the resulting level-specific mean reference waveform.

The time coordinate of the k-th target point was defined as the mean time of the corresponding 10 original samples:

(2) $$t_{k}=0.045+0.1(k-1),\;k=1,2,\ldots ,100$$

where $t_{k}$ is expressed in seconds. Therefore, the resulting target current points were located at 0.045, 0.145, 0.245, …, and 9.945 s. Each target point represented the mean current over a 100 ms interval.

No additional digital filtering was applied. The 10-point block averaging described above was used only for data reduction before pointwise averaging across the five replicate responses; additional smoothing was avoided because it could attenuate the peak current and alter the transient characteristics of the response.

The relationship between the DAC input code and the actual output current of the programmable constant current source was experimentally measured using the Keysight 34470A digital multimeter. The calibration points used for DAC code table generation are listed in Table 1.

The measured output current at a DAC code of zero was 0.016 μA, indicating a small zero code offset. All target current points used in this study were within the experimentally measured output range of 0.016–9.829 μA.

For each target current point, the corresponding DAC input code was calculated using piecewise linear interpolation between two adjacent calibration points. When $I_{i}\leq I_{t\arg et}(k)\leq I_{i+1}$, the unrounded DAC code was calculated as

(3) $$D_{raw}(k)=D_{i}+\frac{I_{t\arg et}(k)-I_{i}}{I_{i+1}-I_{i}}(D_{i+1}-D_{i})$$

where $I_{i}$ and $I_{i+1}$ are two adjacent measured output currents and $D_{i}$ and $D_{i+1}$ are their corresponding DAC input codes. $D_{raw}$ is the unrounded DAC code corresponding to the k-th target current point.

Because the STM32 DAC [17] accepts only integer input codes, the calculated value was rounded to the nearest integer and limited to the valid range of the 12-bit DAC:

(4) $$D(k)=\min[4095,\max(0,round[D_{raw}(k)])]$$

where D(k) is the final DAC input code corresponding to the k-th target current point.

The above calculation was repeated for all 100 target points to generate one level-specific DAC code table for each mean reference waveform. Thus, two 100-point DAC code tables were generated corresponding to control solution levels I and II. During dynamic current reproduction, the STM32 updated the DAC output every 100 ms; therefore, each 100-point output waveform lasted 10 s.

All experimentally measured calibration points, including the full-scale code of 4095, were retained for piecewise interpolation because they represented the actual output current of the circuit. However, because a slight deviation from linearity was observed near the full-scale region, only the data from DAC codes 0–4000 were used for the overall linear regression analysis, while the point at code 4095 was evaluated separately.

### 2.3. System Workflow and Operation

**Figure 3:**
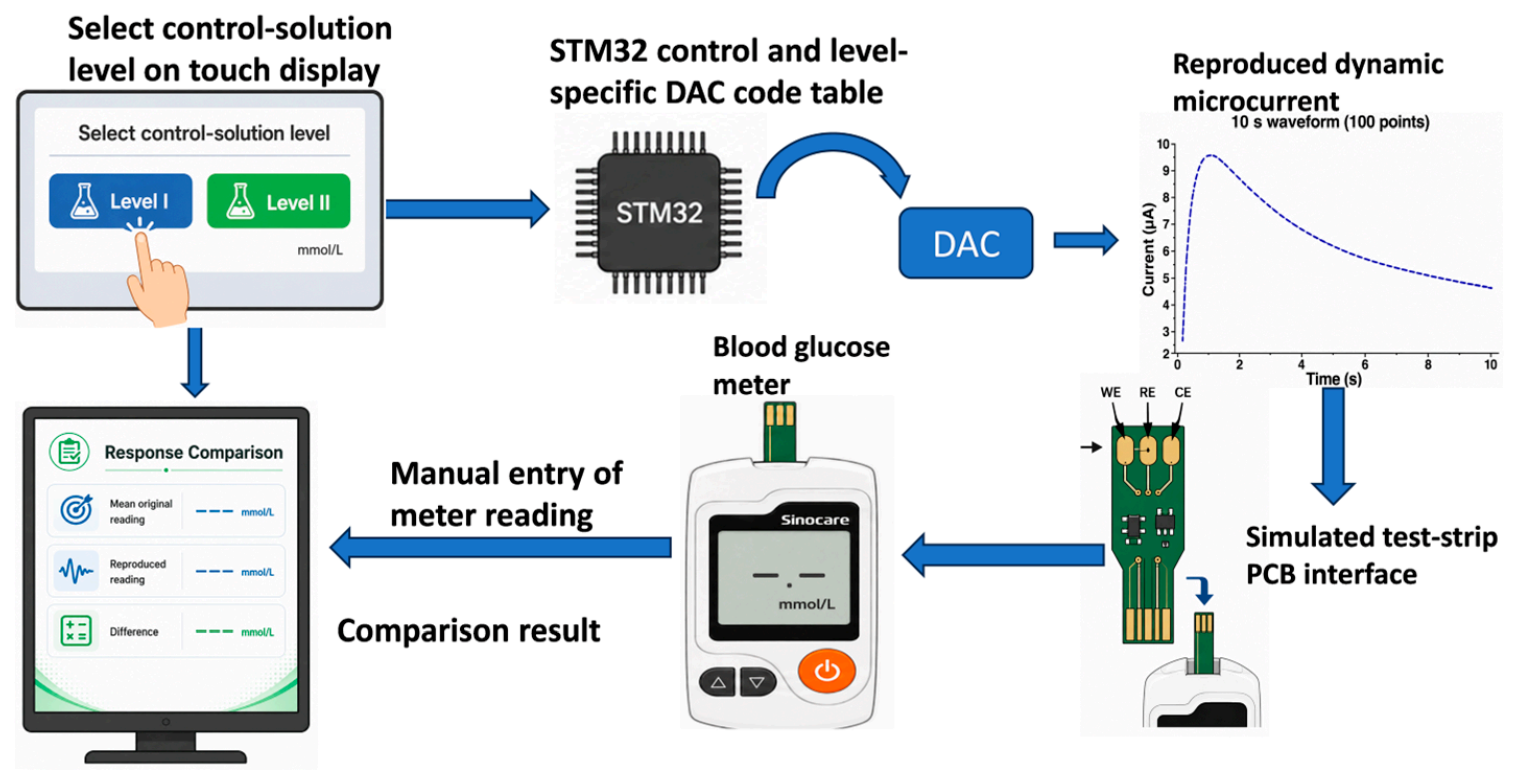
Workflow of the proposed dynamic microcurrent reproduction and electrical response verification system for a blood glucose meter. Arrows indicate the direction of signal flow and data transfer between the functional modules.

Based on the level-specific 100-point DAC code tables derived from the mean reference waveforms in Section 2.2, a portable electrical response verification system was developed to reproduce dynamic microcurrent signals and evaluate blood glucose meter responses. Figure 3 illustrates the complete operating workflow, including control solution level selection, DAC code table execution, dynamic current generation, signal delivery through the simulated test strip PCB interface, meter measurement, and result comparison.

The 4.3-inch X2-series capacitive-touch HMI display (Shenzhen Taojingchi Electronics Co., Ltd., Shenzhen, China) provided two control solution level options corresponding to control solution levels I and II. Each option was linked to the corresponding level-specific 100-point DAC code table derived from the associated mean reference waveform and used to initiate dynamic current reproduction.

After a control solution level was selected, the STM32 sequentially output the DAC codes stored in the corresponding 100-point code table at 100 ms intervals. The DAC and programmable microampere-level constant current source converted the code sequence into a 10 s dynamic microcurrent waveform containing 100 output points.

The reproduced current waveform was delivered to the GA-3 blood glucose meter through the simulated test strip PCB interface, thereby reproducing the principal temporal characteristics of the corresponding mean reference waveform at the meter input. After the meter completed the measurement, its displayed reading was manually recorded. Repeated readings obtained using the reproduced waveform were compared with the corresponding mean original meter reading obtained from the five control solution measurements. The mean difference, relative difference, and short-term repeatability were evaluated using the methods described in Section 2.5.3.

In practical use, source-side and meter-side deviations can be preliminarily distinguished by first independently verifying the reproduced current waveform against the target waveform using a reference current measurement instrument. If the measured waveform deviates from the target, this suggests a source-side deviation; if the waveform agrees with the target but the meter response is abnormal or poorly repeatable, this suggests a meter-side deviation.

### 2.4. Design of the Programmable Microampere-Level Constant Current Source

**Figure 4:**
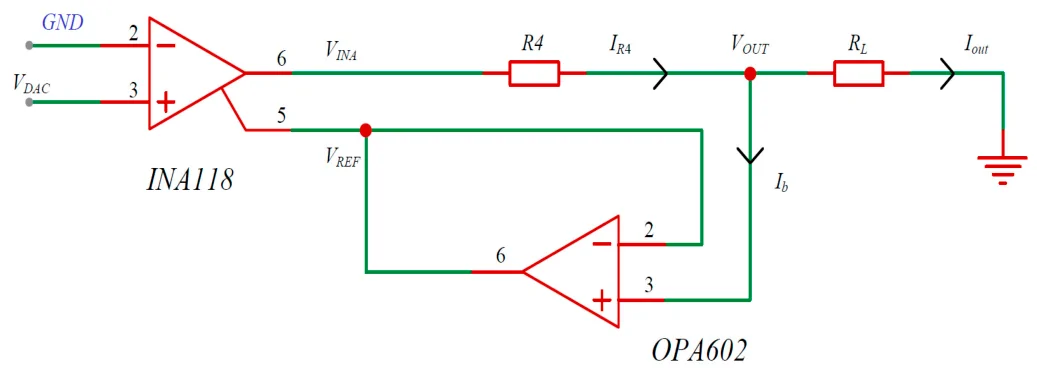
Schematic design of the programmable microampere-level constant current source. The colors are used only to distinguish different circuit elements and connections and do not represent additional physical meanings.

The programmable microampere-level constant current source is the main current output module of the proposed system and is used to convert the DAC output voltage into a controlled microcurrent signal. Previous studies have employed closed-loop constant current source structures based on instrumentation amplifiers to generate programmable microampere-level currents and reduce the effects of input bias current and load grounding [18]. Based on this principle, the circuit developed in this study employs an INA118 instrumentation amplifier (Texas Instruments, Dallas, TX, USA), an OPA602 operational amplifier (Texas Instruments, Dallas, TX, USA), and a precision current setting resistor $R_{4}$. As shown in Figure 4, the DAC output voltage $V_{DAC}$ is applied to the INA118, while the generated output current $I_{out}$ is delivered to the analog test strip interface of the blood glucose meter.

The precision current setting resistor $R_{4}$ had a resistance of 330 kΩ and a tolerance of 0.1%. The gain-setting pins 1 and 8 of the INA118 were left open. According to the INA118 datasheet [19], this configuration corresponds to unity gain:

(5) $$G_{INA}=1$$

The voltage variables used in the following circuit analysis are labeled in Figure 4. Here, $V_{DAC}$ denotes the DAC output voltage applied to the non-inverting input of the INA118, $V_{INA}$ denotes the INA118 output voltage, $V_{REF}$ denotes the voltage at the REF pin of the INA118, and $V_{OUT}$ denotes the voltage at the output side of the current-setting resistor $R_{4}$.

Because the inverting input of the INA118 is connected to ground and the INA118 operates at unity gain, its output voltage can be expressed as

(6) $$V_{INA}=V_{REF}+V_{DAC}$$

The OPA602 is configured as a voltage follower [20]. The node voltage $V_{OUT}$ is applied to the non-inverting input of the OPA602, while the output of the OPA602 is connected to the REF pin of the INA118 and fed back to its inverting input. Therefore,

(7) $$V_{REF}\approx V_{OUT}$$

The current flowing through the precision current-setting resistor $R_{4}$ can therefore be written as

(8) $$I_{R_{4}}=\frac{V_{INA}-V_{OUT}}{R_{4}}=\frac{V_{DAC}+V_{REF}-V_{OUT}}{R_{4}}$$

Using the voltage–follower relationship in Equation (7), the current through $R_{4}$ can be approximated as

(9) $$I_{R_{4}}\approx \frac{V_{DAC}}{R_{4}}$$

According to Kirchhoff’s current law [21], the current through $R_{4}$ is divided into the input bias current $I_{b}$ of the OPA602 and the output current $I_{out}$ delivered to the load:

(10) $$I_{R_{4}}=I_{b}+I_{out}$$

Because the input bias current of the OPA602 is much smaller than the microampere-level output current [20], its effect can be neglected. Therefore,

(11) $$I_{out}\approx I_{R_{4}}$$

Combining Equations (9) and (11), the output current is mainly determined by the DAC output voltage $V_{DAC}$ and the resistance of $R_{4}$. With $R_{4}=330\;k\Omega$ and $V_{DAC}$ varying from 0 to 3.3 V, the designed output current range is approximately 0–10 μA. This value represents the theoretical design range; the actual output range and output characteristics were experimentally evaluated and are reported in Section 3.1.

The STM32 DAC has a 12-bit resolution. Based on the designed current range of 0–10 μA, the theoretical current step is

(12) $$\Delta I_{theoretical}=\frac{10\mu A}{2^{12}}\approx 0.0024\mu A$$

Within the allowable operating range of the circuit, the OPA602 feedback path helps reduce the influence of load voltage variations on the output current. The load capability of the constant current source was experimentally evaluated as described in Section 2.5.

An LM27762 bipolar power module (Texas Instruments, Dallas, TX, USA) was used to provide ±5 V analog supplies for the INA118 and OPA602 [22]. In the PCB layout, the analog and digital power and ground regions were separated, and AGND and DGND were connected at a single point through a 0 Ω resistor to reduce digital interference in the analog current output circuit [23].

### 2.5. Experimental Setup and Evaluation Metrics

**Figure 5:**
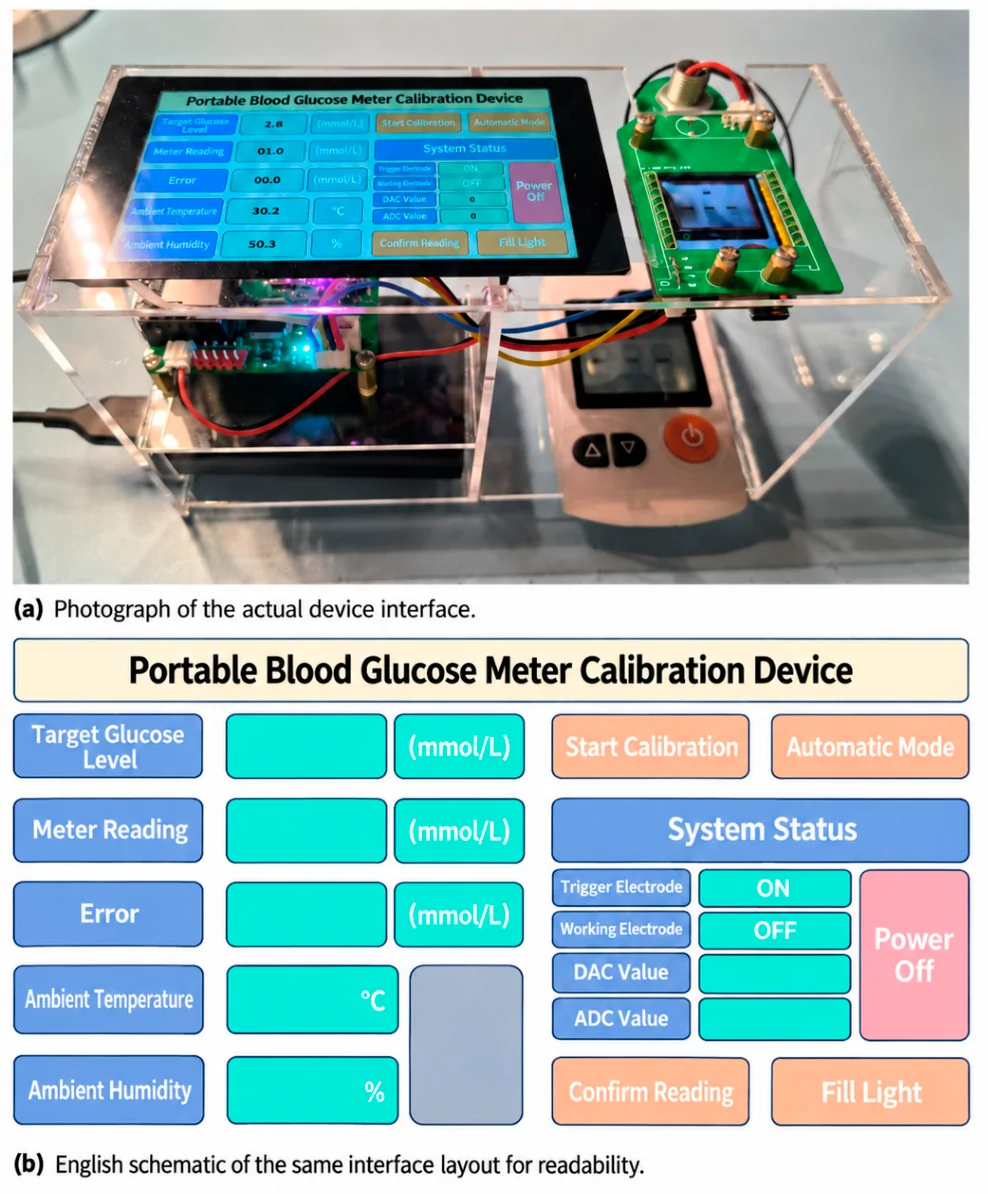
(**a**) Photograph of the developed portable electrical response verification system and the GA-3 blood glucose meter, with the main on-screen labels translated into English for readability. (**b**) English schematic of the same interface layout for improved readability.

The developed portable electrical response verification system and the GA-3 blood glucose meter are shown in Figure 5. A Keysight 34470A seven-and-a-half-digit digital multimeter was used to measure the output current during the calibration of the constant current source and the evaluation of dynamic waveforms.

#### 2.5.1. Constant Current Source Evaluation

The load regulation performance of a voltage-controlled current source is closely related to its output impedance; a higher output impedance typically reduces the dependence of the output current on changes in the load resistance [24]. Therefore, in this study, the load performance of the fabricated constant current source was evaluated under load conditions of 10, 20, 51, and 80 kΩ. For the load regulation test, DAC input codes of 0, 500, 1000, 2000, 3000, and 4000 were evaluated under each load condition.

The actual DAC-controlled output performance was then evaluated using the fabricated PCB. The tested DAC input codes were 0, 400, 800, 1200, 1600, 2000, 2400, 2800, 3200, 3600, 4000, and 4095. The DAC codes were applied in both forward and reverse directions. The output current at each code was measured three times using the Keysight 34470A digital multimeter, and the mean value was used for analysis.

Linear regression was performed using the data from DAC codes 0–4000. The full-scale point at code 4095 was evaluated separately because a small deviation from linearity was observed near the upper end of the output range. The coefficient of determination, $R^{2}$, was used to evaluate the linear relationship between the DAC input code and the output current.

The hysteresis error was calculated by comparing the forward- and reverse-sweep currents measured at the same DAC input code:

(13) $$E_{hys}=\frac{\max_{D}|I_{F}(D)-I_{R}(D)|}{I_{FS}}\times 100\%$$

where $I_{F}(D)$ and $I_{R}(D)$ are the mean output currents measured during the forward and reverse sweeps at DAC input code D, respectively, and $I_{FS}$ is the measured full-scale output current.

#### 2.5.2. Dynamic Current Reproduction Evaluation

For each control solution level, the level-specific mean reference waveform was used as a fixed target waveform for dynamic current reproduction. The same 100-point DAC code table was executed in five repeated runs using the programmable microampere-level constant current source. During each run, the DAC codes were output at 100 ms intervals, and the output current was recorded using a Keysight 34470A digital multimeter. The multimeter acquisition interval was set to 100 ms (10 Hz), matching the DAC update interval. Each reproduction run corresponded to one GA-3 meter response measurement, as described in Section 2.5.3.

For each reproduction, the recorded current sequence included data before and after the waveform output period. The first point after the clear transition from the pre-output baseline was defined as the start of the reproduced waveform. A continuous sequence of 100 measured points was then retained and aligned point by point with the corresponding target waveform.

The numerical differences between the target and measured waveforms were evaluated using the mean absolute error (MAE) and root mean square error (RMSE) [25]:

(14) $$MAE=\frac{1}{N}\sum_{k=1}^{N}\left|I_{meas}(k)-I_{t\arg et}(k)\right|$$

(15) $$RMSE=\sqrt{\frac{1}{N}\sum_{k=1}^{N}{\left[I_{meas}(k)-I_{t\arg et}(k)\right]}^{2}}$$

where $I_{t\arg et}(k)$ and $I_{meas}(k)$ are the target and measured currents at point k, respectively, and N=100.

Waveform similarity was evaluated using the Pearson correlation coefficient r, which was calculated from 100 pairs of target current points and measured current points. Because the waveform points were continuously sampled and not treated as independent observations, r was used only as a descriptive measure of waveform shape similarity, and no correlation-based significance test was performed [26].

For each control solution level, MAE, RMSE, and Pearson r were calculated separately for each of the five reproduced waveforms relative to the corresponding target waveform. In addition, the peak current of each reproduced waveform was extracted, and the mean, sample standard deviation (SD), and coefficient of variation (CV) were calculated to evaluate the repeatability of dynamic current generation. The pointwise mean of the five reproduced waveforms was calculated for graphical comparison with the target waveform only.

#### 2.5.3. Blood Glucose Meter Response Evaluation

For each control solution level, the five GA-3 meter readings corresponding to the five dynamic reproduction runs described in Section 2.5.2 were used for meter response evaluation. One displayed meter reading was recorded for each reproduction run. The mean and sample standard deviation were used to summarize the five readings, while the coefficient of variation was used to describe their relative dispersion and short-term repeatability [27,28]. The mean reproduced meter reading was then compared with the mean original meter reading obtained from the five corresponding control solution measurements at the same control solution level.

The mean reproduced reading was calculated as

(16) $$\bar{G}=\frac{1}{n}\sum_{i=1}^{n}G_{i}$$

The sample standard deviation was calculated as

(17) $$s=\sqrt{\frac{\sum_{i=1}^{n}{\left(G_{i}-\bar{G}\right)}^{2}}{n-1}}$$

The mean difference was defined as

(18) $$\Delta G=\bar{G}-{\bar{G}}_{orig}$$

The relative difference was calculated using the absolute value of the mean difference:

(19) $$E_{rel}=\frac{\left|\bar{G}-{\bar{G}}_{orig}\right|}{{\bar{G}}_{orig}}\times 100\%$$

The coefficient of variation was calculated as

(20) $$CV=\frac{s}{\bar{G}}\times 100\%$$

where ${\bar{G}}_{orig}$ is the mean of the five original meter readings obtained from the corresponding control solution level, $G_{i}$ is the reading obtained from the i-th reproduced waveform test, $\bar{G}$ is the mean of the five reproduced readings, s is the sample standard deviation of the reproduced readings, and n=5.

## 3. Results

### 3.1. Performance of the Constant Current Source

**Figure 6:**
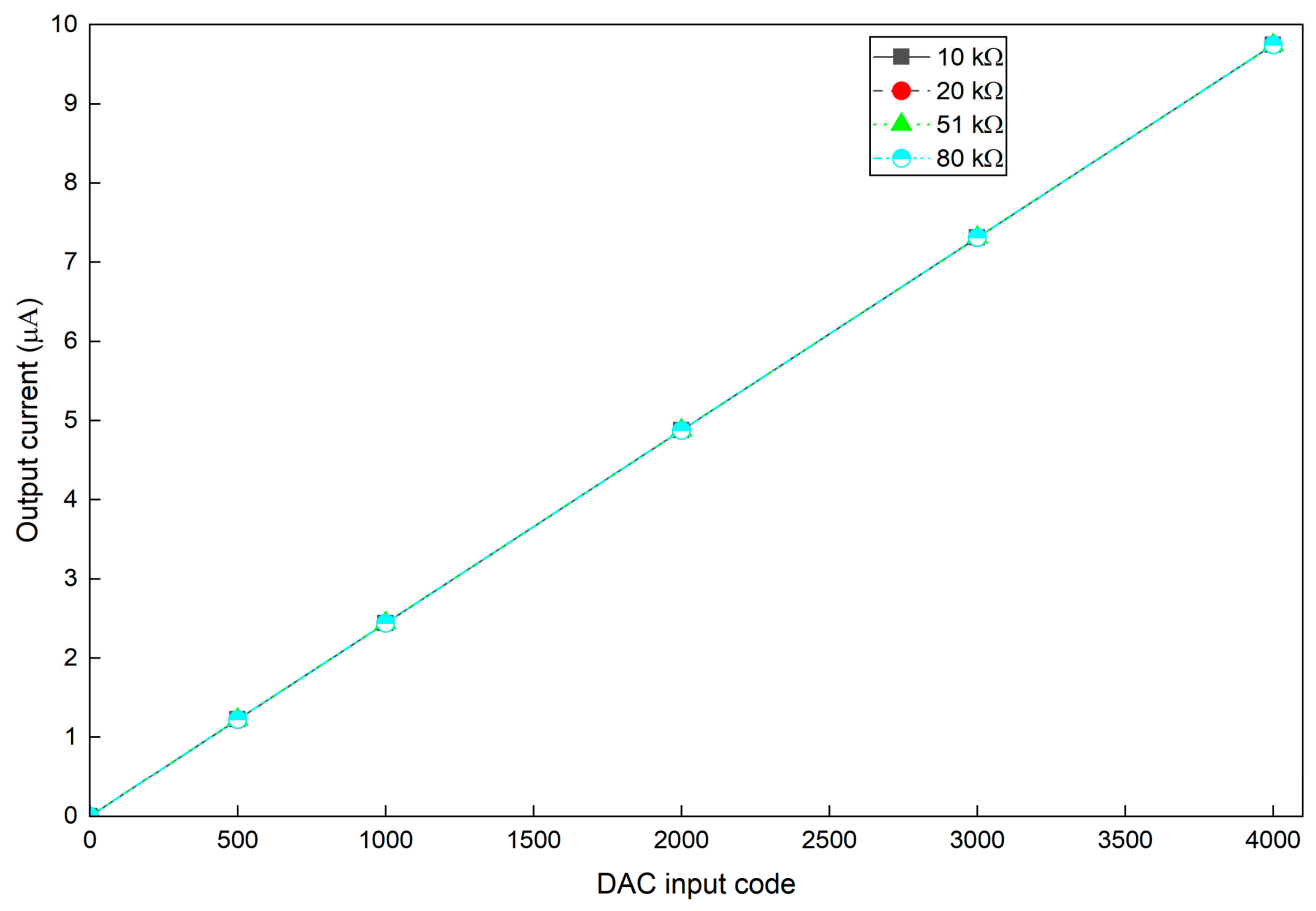
Measured output current as a function of DAC input code under load resistances of 10, 20, 51, and 80 kΩ.

The output performance of the programmable microampere-level constant current source was first evaluated under different load conditions. Four load resistances of 10, 20, 51, and 80 kΩ were tested. For each load, the DAC input codes were set to 0, 500, 1000, 2000, 3000, and 4000, and the corresponding output current was measured. The results are shown in Figure 6.

As shown in Figure 6, the output current increased approximately linearly with the DAC input code under all four load conditions. At DAC code 0, the measured output current was approximately 0.016 μA for all four loads. Across the tested nonzero DAC input codes, the maximum absolute difference in output current among the four load conditions was 0.003 μA, occurring at DAC code 3000. The maximum relative variation was approximately 0.16%, occurring at DAC code 500. These results indicate that the designed constant current source maintained stable programmable current output over the tested load range of 10–80 kΩ.

**Figure 7:**
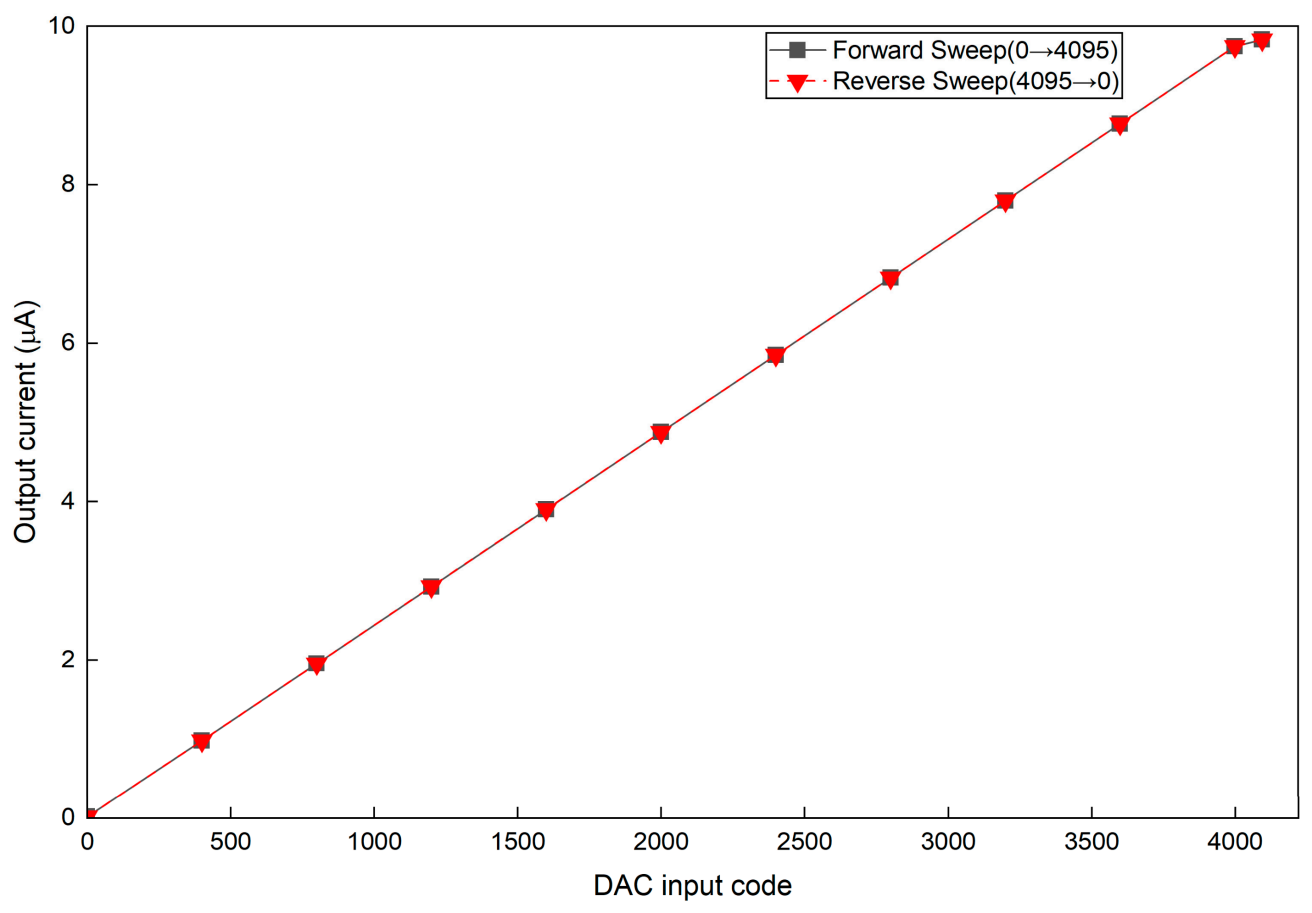
Output characteristics of the microampere-level constant current source under DAC control. The curves represent the output current measured during forward and reverse DAC input code sweeps.

The results of the forward and reverse scans are shown in Figure 7; each data point represents the average of three measurements.

As shown in Figure 7, the output current increased almost linearly with the DAC input code. During the forward sweep, the DAC input code increased from 0 to 4095, and the output current increased from 0.016 μA to 9.829 μA. During the reverse sweep, the output current decreased from 9.829 μA to 0.016 μA. The forward and reverse sweep curves showed good consistency over the entire DAC input range, indicating little dependence of the output current on the scanning direction.

To further evaluate the relationship between the DAC input code and the output current, linear regression analysis was performed. Because slight nonlinear behavior may occur near the DAC full-scale region, the data points from 0 to 4000 were selected for linear fitting, while the maximum code value of 4095 was evaluated separately.

The fitting equation for the forward sweep was

(21) $$I_{forward}=0.00243377x+0.00927$$

with a coefficient of determination of

(22) $$R^{2}=0.99999$$

The fitting equation for the reverse sweep was

(23) $$I_{reverse}=0.00243389x+0.00941$$

with

(24) $$R^{2}=0.99999$$

where x represents the DAC input code and $I_{forward}$ and $I_{reverse}$ represent the corresponding output currents (μA) for the forward and reverse sweeps, respectively. The high coefficients of determination demonstrate a strong linear relationship between the DAC input code and the output current within the main operating range.

The hysteresis characteristic was further evaluated by comparing the output current values obtained from the forward and reverse sweeps at the same DAC input code. The maximum current difference was approximately 0.001 μA. Compared with the full-scale output current of 9.829 μA, the corresponding maximum hysteresis error was approximately 0.01%.

At the maximum DAC code of 4095, the measured output current was 9.829 μA for both forward and reverse sweeps. The slight deviation between the measured results and the linear fitting results near the full-scale region may be attributed to the output swing limitation of the analog circuit and the non-ideal characteristics of electronic components.

Overall, the experimental results demonstrate that the designed microampere-level constant current source can generate a linear current output controlled by DAC input codes with a small hysteresis error. This performance provides a reliable hardware basis for generating dynamic microcurrent signals in the proposed electrical response verification system.

### 3.2. Repeatability and Agreement of Dynamic Current Reproduction

**Figure 8:**
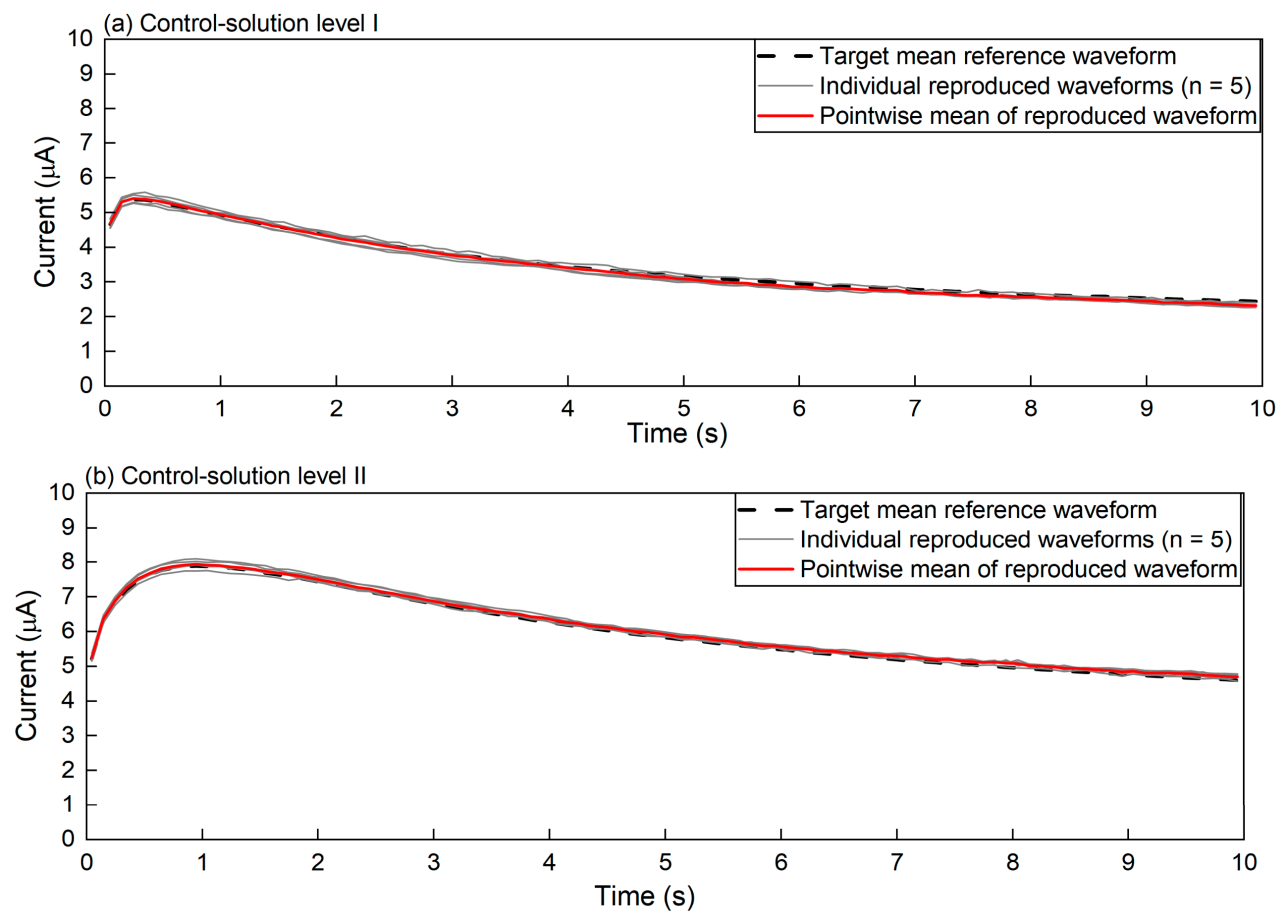
Repeated dynamic current reproduction of the level-specific mean reference waveforms for (**a**) control solution level I and (**b**) control solution level II. The black dashed curve represents the target mean reference waveform derived from the original control solution measurements, the gray curves represent five independently reproduced waveforms, and the red curve represents their pointwise mean.

The level-specific mean reference waveforms obtained for control solution levels I and II were used as fixed target waveforms for dynamic current reproduction. For each level, the same 100-point DAC code table was executed in five repeated runs using the programmable microampere-level constant current source. Figure 8 compares the five reproduced waveforms and their pointwise mean with the corresponding target waveform for each control solution level.

As shown in Figure 8, the five reproduced waveforms for both control solution levels exhibited similar temporal profiles and closely followed the corresponding target waveforms throughout the 10 s output period. The pointwise mean of the five reproduced waveforms also showed good agreement with the target waveform, indicating stable repeated generation of the dynamic microcurrent responses. The quantitative results are summarized in Table 2.

As shown in Table 2, the peak current CV values were 2.52% and 1.59% for levels I and II, respectively. The mean MAE and RMSE values were 0.0929 and 0.1045 μA for level I and 0.0918 and 0.1012 μA for level II, while the corresponding mean Pearson correlation coefficients were 0.9993 and 0.9995. Taken together, these metrics indicate good numerical agreement, waveform shape similarity, and short-term reproduction repeatability. Because correlation alone does not establish numerical agreement [29], Pearson r was interpreted together with MAE and RMSE.

### 3.3. Blood Glucose Meter Response to Reproduced Current Waveforms

For each control solution level, the five dynamic reproduction runs evaluated in Section 3.2 yielded five corresponding GA-3 meter readings. These readings were used to evaluate meter response consistency under repeated electrical stimulation. The mean reproduced reading, sample standard deviation (SD), coefficient of variation (CV), mean difference, and relative difference were calculated with respect to the mean original meter reading obtained from the five corresponding control solution measurements. The results are summarized in Table 3.

The reproduced meter readings are reported as mean ± sample standard deviation (SD) based on five repeated measurements (n = 5), while the mean original meter reading was calculated from the five corresponding control solution measurements. Because individual meter readings were displayed to 0.1 mmol/L, the mean values and SDs calculated from repeated measurements were reported to two decimal places in mmol/L for consistent statistical presentation. Values in mg/dL were converted from mmol/L using a conversion factor of 18. The mean difference, relative difference, and coefficient of variation (CV) were calculated as described in Section 2.5.3.

As shown in Table 3, the reproduced meter responses were close to the corresponding mean original readings, with relative differences of 1.70% and 1.18% and CV values of 1.81% and 1.29% for levels I and II, respectively. These results indicate good short-term response consistency under repeated electrical stimulation.

## 4. Discussion

The proposed system showed stable programmable microampere-level output and repeatable reproduction of experimentally acquired test strip current responses. Because the DAC code tables were generated from the experimentally measured DAC code–current relationship rather than solely from the ideal transfer equation, the reproduced waveforms accounted for the actual characteristics of the analog circuit. Across load resistances of 10–80 kΩ, the maximum absolute current difference was 0.003 μA, with a maximum relative variation of approximately 0.16%, supporting stable operation of the current source for dynamic waveform reproduction.

Previous evaluations of point-of-care blood glucose meters have assessed analytical agreement and precision through comparison with reference laboratory methods [30]. At the sensor level, amperometric glucose biosensors generate glucose-dependent current responses, demonstrating the fundamental relationship between glucose sensing and electrochemical current signals [31]. The current response of electrochemical blood glucose test strips is time-dependent and influenced by the enzymatic reaction and transport of electroactive mediators within the reagent membrane [32]. Therefore, a single constant current value cannot fully represent the temporal characteristics of the electrical input presented to the meter. This does not imply that the meter directly uses the entire waveform for glucose calculation; rather, preserving the principal temporal profile avoids assumptions about the meter’s undisclosed internal processing algorithm. For each control solution level, five original current responses were used to establish one level-specific mean reference waveform, with peak current CV values of 2.55% and 3.50% for levels I and II, respectively. Five repeated reproductions yielded mean Pearson correlation coefficients of 0.9993 and 0.9995 and peak current CV values of 2.52% and 1.59%, respectively. Together with the low MAE and RMSE values, these results indicate good waveform agreement and short-term reproduction repeatability. Residual differences may arise from DAC quantization, analog circuit settling, measurement noise, and timing mismatch between DAC updating and current acquisition.

The same five dynamic reproduction runs yielded five corresponding GA-3 meter readings for each control solution level. The mean reproduced readings were 4.62 ± 0.08 mmol/L (83.2 ± 1.5 mg/dL) and 10.08 ± 0.13 mmol/L (181.4 ± 2.3 mg/dL), with CV values of 1.81% and 1.29% for levels I and II, respectively. The relative differences from the mean original readings were 1.70% and 1.18%, supporting the feasibility of short-term electrical response verification. However, this agreement should not be interpreted as independent verification of glucose measurement accuracy because the mean reference waveform and mean original meter reading for each level were derived from the same five original control-solution measurements. Control solution testing [5], ISO 15197/reference-based evaluation [33,34], and the proposed method are compared in Table 4 using common functional dimensions rather than direct numerical performance metrics.

As summarized in Table 4, the representative approaches address different evaluation objectives and are therefore compared using common functional dimensions rather than direct numerical performance metrics. Control solution testing evaluates the combined meter–strip–reagent system [5], whereas ISO 15197/reference-based evaluations assess the analytical performance of the complete measurement system, including system accuracy and precision [33,34]. In contrast, the proposed method focuses specifically on the meter electrical input–response path by reproducing a characterized dynamic current–time waveform. Accordingly, the revised comparison emphasizes differences in evaluation scope, the availability of a directly controlled and pre-characterized electrical stimulus, the requirement for a new strip/reagent reaction, and intended use rather than ranking the approaches by numerical performance.

The distinguishing capability of the proposed method therefore does not lie in achieving higher analytical accuracy than conventional or ISO-based approaches. Rather, once a level-specific mean reference waveform and its corresponding DAC code table have been established, the same characterized electrical stimulus can be repeatedly applied during bench top verification. This provides a controlled condition for evaluating waveform reproduction performance and short-term meter response repeatability without requiring a new strip/reagent reaction for each repeated electrical test. The proposed method does not evaluate strip/reagent performance or analytical accuracy against an independent reference method and should therefore be regarded as complementary to conventional control solution and ISO/reference-based evaluations.

The proposed method nevertheless has several limitations. It does not assess reagent activity, sample-related effects, hematocrit or other interferences, user handling errors, or analytical accuracy against an independent reference glucose method; therefore, it should be regarded as complementary to conventional control solution testing and ISO 15197:2013-based evaluation [5,34]. Moreover, five original control solution measurements were used for reference waveform acquisition, and five dynamic reproduction runs were used for both waveform reproduction and corresponding meter response evaluation to assess short-term repeatability. Validation was nevertheless limited to one blood glucose meter model, one test strip lot, and two control solution levels. Future work should include larger sample sizes, additional meter models, multiple test strip lots and control solution levels, and long-term output stability evaluation.

## 5. Conclusions

This study developed a portable electrical response verification system for blood glucose meters based on dynamic microcurrent reproduction. For each of two control solution levels, five independent 1000-point test strip current responses were individually reduced to 100 points and then averaged point by point to establish one level-specific mean reference waveform. Each mean reference waveform was converted into a 100-point DAC code table for STM32-controlled reproduction. The programmable current source provided a measured output range of 0.016–9.829 μA and maintained stable output over load resistances of 10–80 kΩ, with a maximum relative variation of approximately 0.16%.

Each level-specific mean reference waveform was reproduced in five runs using the same DAC code table. The mean Pearson correlation coefficients were 0.9993 and 0.9995, with peak current CV values of 2.52% and 1.59% for levels I and II, respectively. The corresponding GA-3 meter readings differed from the mean original readings by 1.70% and 1.18%, with meter response CV values of 1.81% and 1.29%. These results demonstrate the feasibility of the proposed system for controlled dynamic electrical response verification and short-term repeatability assessment.

The proposed method is intended to complement, rather than replace, conventional control solution testing or reference-based analytical evaluation. Once a level-specific mean reference waveform and its corresponding DAC code table have been established, the same characterized electrical stimulus can be repeatedly applied during subsequent bench top electrical verification without requiring a new test strip/control solution reaction for each run. This advantage is limited to engineering and laboratory verification and does not reduce the need for disposable test strips in routine patient glucose monitoring. Broader validation across additional meters, test strip lots, control solution levels, and longer-term operation is warranted.

## 6. Patents

A patent related to the portable blood glucose meter calibration device described in this study has been filed and is currently under review.

## Figures and Tables

**Table 1:** Measured DAC code–current calibration points used for DAC code table generation.

DAC Input Code	Measured Output Current (μA)
0	0.016
400	0.980
800	1.954
1200	2.928
1600	3.902
2000	4.876
2400	5.850
2800	6.824
3200	7.798
3600	8.771
4000	9.746
4095	9.829

**Table 2:** Quantitative evaluation of the agreement and repeatability of dynamic current reproduction for the two control solution levels.

Control Solution Level	Peak Current ± SD (μA)	Peak Current CV (%)	MAE (μA)	RMSE (μA)	Pearson r
I	5.412 ± 0.136	2.52	0.0929 ± 0.0212	0.1045 ± 0.0161	0.9993 ± 0.0002
II	7.945 ± 0.126	1.59	0.0918 ± 0.0483	0.1012 ± 0.0482	0.9995 ± 0.0002

**Table 3:** Comparison of the mean original meter readings with repeated GA-3 meter readings obtained during dynamic current reproduction.

Control Solution Level	Mean Original Meter Reading, mmol/L (mg/dL)	Mean Reproduced Meter Reading ± SD, mmol/L (mg/dL)	Mean Difference, mmol/L (mg/dL)	Relative Difference (%)	CV (%)
I	4.70 (84.6)	4.62 ± 0.08 (83.2 ± 1.5)	−0.08 (−1.4)	1.70	1.81
II	10.20 (183.6)	10.08 ± 0.13 (181.4 ± 2.3)	−0.12 (−2.2)	1.18	1.29

**Table 4:** Functional comparison of representative approaches for blood glucose meter evaluation.

Approach	Test Input	Evaluation Scope	Directly Controlled, Pre-Characterized Electrical Stimulus	New Strip/Reagent Reaction Required	Primary Intended Use
Control solution testing [5]	Control solution + test strip	Combined meter–strip–reagent system	Not the intended function	Yes	Routine functional quality control
ISO 15197/reference-based evaluation [33,34]	Blood samples + reference measurement	Complete measurement system and analytical performance	Not the intended function	Yes	Analytical accuracy and precision evaluation
Proposed dynamic electrical response verification (this study)	Characterized dynamic current–time waveform	Meter electrical input–response path	Yes	No, once the reference waveform has been established	Bench top engineering verification and short-term repeatability assessment

## Data Availability

The data supporting the findings of this study are available from the corresponding author upon reasonable request.

## Abbreviations

The following abbreviations are used in this manuscript:

DAC	Digital-to-Analog Converter
PCB	Printed Circuit Board
SD	Standard Deviation
STM32	STMicroelectronics 32-bit microcontroller
MAE	Mean Absolute Error
RMSE	Root Mean Square Error
CV	Coefficient of Variation
SMBG	Self-Monitoring of Blood Glucose
CGM	Continuous Glucose Monitoring
POCT	Point-of-Care Testing
AGND	Analog Ground
DGND	Digital Ground
